# Kaleidoscopic protein–protein interactions in the life and death of ataxin-1: new strategies against protein aggregation^☆^

**DOI:** 10.1016/j.tins.2014.02.003

**Published:** 2014-04

**Authors:** Cesira de Chiara, Annalisa Pastore

**Affiliations:** 1 National Institute for Medical Research (NIMR), Medical Research Council (MRC), The Ridgeway, London NW7 1AA, UK; 2 Department of Clinical Neurosciences, King's College London, Denmark Hill Campus, London, UK

**Keywords:** misfolding diseases, polyglutamine, protein aggregation, protein–protein interactions

## Abstract

Understanding how proteins protect themselves from aberrant aggregation is of primary interest for understanding basic biology, protein biochemistry, and human disease. We discuss the paradigmatic example of ataxin-1 (Atx1), the protein responsible for neurodegenerative spinocerebellar ataxia type 1 (SCA1). This disease is part of the increasing family of pathologies caused by protein aggregation and misfolding. We discuss the importance of protein–protein interactions not only in the nonpathological function of Atx1 but also in protecting the protein from aggregation and misfolding. The lessons learned from Atx1 may lead to a more general understanding of the cell's protective strategies against aggregation. The obtained knowledge may suggest a new perspective for designing specific therapeutic strategies for the cure of misfolding diseases.

## Highlights

• Ataxin-1 (Atx1) is the protein responsible for spinocerebellar ataxia type 1 (SCA1).
• Normal function and anomalous aggregation are competing pathways.
• Protein–protein interactions protect Atx1 from aggregation and misfolding.
• This knowledge can be exploited in drug development.

## Cellular mechanisms of protection from protein aggregation

The increasing realization that protein aggregation plays a predominant role in the development of a number of diseases parallels an increasing interest in the mechanisms that protect proteins from this often unwanted phenomenon. Although much has been said about these protection mechanisms, and several possible strategies that the cell may take in combating protein aggregation have been suggested [1–4], our knowledge of the topic remains limited. One of the most interesting hypotheses is that interactions with other cellular partners could have a leading role in preventing protein aggregation [3,5]. Several examples seem to support this hypothesis. It has, for instance, been observed that binding to ligands, including nucleic acids, prevents misfolding of the oncogene p53 [4]. Similarly, the protein ataxin-3 has been found to be protected from aggregation when interacting with the natural cellular partner ubiquitin [6]. These examples in turn suggest that normal function and aberrant aggregation are competing pathways [7].

In this review, we discuss the paradigmatic example of Atx1. This protein provides a unique illustration of how protein–protein interactions can sort the ‘fate’ of a protein. Atx1 is responsible for SCA1 (OMIM #164400) [8] (for a definition of ataxia, see the Glossary). SCA1 is a late onset autosomal dominant neurodegenerative disorder characterized by cerebellar ataxia and associated with varying degrees of oculomotor abnormalities, pyramidal and extrapyramidal features, peripheral neuropathy, and cognitive impairment [9]. Until a link with disease was established, Atx1 had largely been overlooked. However, over the last decade or so, a substantial amount of research into the structure and function of Atx1 has started to provide a clearer picture of the cellular role of this protein [10,11].

In the following sections, we review our current knowledge of Atx1 and place these observations within the context of the protection mechanisms against aggregation. We show how the normal function of the protein, together with its unusual structural properties, determine its cellular function/dysfunction and how aggregation is mediated by multiple regions acting in cooperation. We believe that this example may open a new perspective for the study of SCA1 and other misfolding diseases and eventually suggest a strategy for a specific cure.

## Atx1 is a member of the polyglutamine expansion diseases

**Figure 1:**
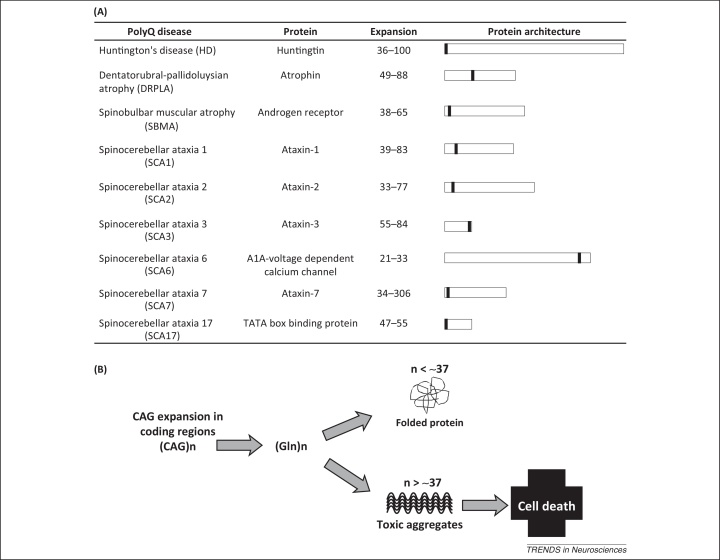
Expansion of a polyglutamine (polyQ) tract in specific proteins is associated with neurodegeneration. **(A)** A list of the currently known polyQ diseases with protein name, pathological threshold, and protein architecture. The position of polyQ in the sequence is indicated as a black rectangle on the protein schematic representation. **(B)** Schematic representation of the disease mechanism. When the repeat number is lower than a threshold (∼37 repeats), the proteins are correctly folded and functional; when it is above the threshold, the carrier proteins aggregate and misfold with consequent cell toxicity and death.

Atx1 is, together with the above mentioned ataxin-3, a member of the family of proteins that contain a polyglutamine (polyQ) tract and are implicated in genetic neurodegenerative diseases [12] (Figure 1A). These pathologies are caused by the anomalous expansion of a polymorphic tract of polyQ, which, when above a threshold of approximately 37 repeats, causes aggregation that is ultimately associated with cell toxicity and neuronal death [12] (Figure 1B). Although rare and diversified in symptoms, these diseases are dominant and currently incurable. Several lines of evidence suggest that polyQ expansion is the necessary event for disease development: attachment of a polyQ stretch to an otherwise healthy protein is sufficient to cause toxicity; interruption of the polyQ tract by even one non-glutamine amino acid appreciably slows down disease and the age at disease onset correlates inversely with the number of uninterrupted polyQ repeats [13]. It has however been recognized that, in addition to polyQ, other regions significantly contribute to the aggregation process. An increasing interest in studying Atx1 stems not only from the desire of finding a cure for SCA1 but also because, being one of the small members of the polyQ family, Atx1 is an excellent model for understanding the behavior of the whole family [14].

## Atx1 contains intrinsically unfolded regions and a chameleon domain

**Figure 2:**
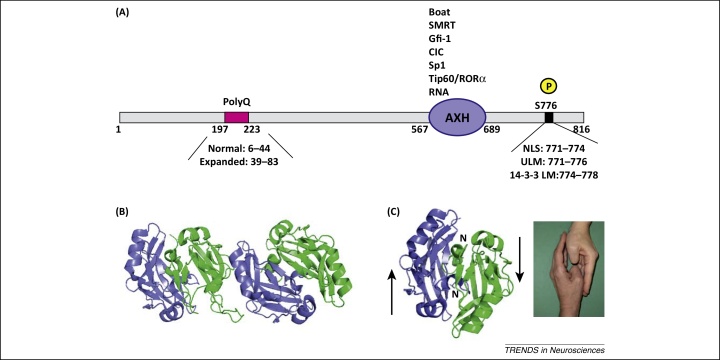
The structure of Atx1. **(A)** A schematic representation of the Atx1 architecture. The protein is represented as a horizontal bar on which the position of the polyQ motif, the AXH domain and S776 are indicated. The interactions formed with various cellular partners are also listed near the interacting motif. **(B)** The crystal structure of the AXH domain (PDB accession code 1oa8, [21]). In the crystallographic asymmetric unit, there are two dimers, which form a dimer of dimers (indicated in the figure with alternated colors). **(C)** Structure of the dimer rotated by 45 degrees around the axis perpendicular to the plane as compared to the view in (B). The overall arrangement is antiparallel with the termini being sandwiched in the dimer interface like in two touching left hands. The symmetry is however incomplete and the individual protomers differ by local details. Most of the differences are grouped at the N termini. Abbreviations: Atx1, ataxin-1; CIC, Capicua; NLS, nuclear localization signal; PDB, Protein Data Bank; polyQ, polyglutamine; UHM, U2AF (U2 auxiliary factor) homology motif; ULM, UHM ligand motif.

Atx1 is a predominantly nuclear, ubiquitous protein well conserved in vertebrates [15]. Human Atx1 is 816 amino acids long although its length can increase depending on the length of the polyQ tract, which varies from six to circa 44 glutamine repeats in the normal population and up to about 83 uninterrupted glutamines in SCA1 patients [10,12,13] (Figure 2A).

Prediction of the structure of Atx1 based on the amino acid sequence indicates that Atx1 is predominantly an intrinsically unstructured protein [14]. The only globular domain present in the protein, named AXH (SMART SM00536), is an independently folded motif of approximately 120 amino acids that was identified on the bases of sequence conservation across species and secondary structure prediction [16]. According to the human Atx1 sequence (UniProtKB/Swiss-Prot No. P54253 +1), this domain spans residues 567–689 [16]. The long N-terminal region 1–566 contains the low complexity polymorphic polyQ tract starting at residue 197 and one of the two experimentally verified phosphorylation sites [14]. The polyQ stretch is expected to be unstructured as shown by circular dichroism (CD) and nuclear magnetic resonance (NMR) spectroscopy [17]. The C terminus (residues 690–816) is also predicted to be unstructured and contains several linear motifs, either clustered in proximity or including the phosphorylable S776, which are extremely important for protein localization [15,18] and recognition of biological partners [19,20].

The intrinsic tendency of Atx1 to degradation and aggregation even when in its non-expanded form has so far hampered the possibility of expressing and purifying the recombinant full-length protein in an intact mono-disperse form suitable for a structural characterization of its conformational ensemble. However, it was possible to produce the AXH domain in isolation and to investigate its structure both in the crystal and in solution. The crystal structure revealed the open barrel typical of an oligonucleotide-binding (OB) fold, a structural motif involved in nucleic acid and protein recognition [21]. The crystal asymmetric unit contains a dimer of dimers (Figure 2B). The individual protomers are arranged in an antiparallel fashion with the N termini sandwiched between the dimer interface. The dimer structure resembles two left hands (the protomers) touching each other in an antiparallel fashion so that the two thumbs (corresponding to the N termini) are close in space and parallel (Figure 2C). The protomers are nonsymmetric having the first approximately 20 N-terminal amino acids of the individual protomers adopting different conformations and thus defining asymmetric dimer interfaces. The unusually variable structure of the N terminus of the domain makes the AXH a unique case of a protein ‘chameleon’, that is, a protein that is able to adopt distinct conformations [21,22]. The presence of a dimeric AXH domain also in solution was demonstrated by analytical ultracentrifugation studies, which showed that the predominant species in the low micromolar range of concentrations is dimeric, although in co-presence with higher molecular species [16,21]. In agreement with these data, small angle X-ray scattering (SAXS) confirmed the existence of a complex equilibrium between monomeric, dimeric, and tetrameric species in solution [22]. Taken together, these findings indicate a strong tendency of the AXH domain to dimerize and suggest that the dimerization observed for the AXH could account for the known self-association of the full-length protein observed in the cell [23].

## In search of Atx1 function: a dual role in transcription and RNA metabolism

Not long after the *SCA1* gene was identified [24], it was discovered that expanded Atx1 has to enter the nucleus of Purkinje cells to become pathogenic [15]. Subsequently, analysis of a transgenic mouse model for *SCA1* showed that gene expression is dysregulated at a very early stage of the disease, when neurological hallmarks are still undetectable [25,26]. Several transcriptional corepressors were also identified as modulators of the Atx1-mediated eye phenotype in a genetic screen in *Drosophila* [27]. Overall, these observations suggested that Atx1 is involved in transcription regulation and that SCA1 pathogenesis could be the result of alterations in gene expression, rather than or in addition to the gain of function mechanism triggered by polyQ expansion.

Using a general read-out assay for repression of transcription it was shown that both full-length Atx1 and the isolated AXH domain repress transcription when tethered to DNA [28,29]. However, interaction with DNA must be mediated by other transcription factors because crosslinking experiments excluded a direct binding between Atx1 and DNA [29].

Atx1 has been described as modulating the function of several transcriptional regulators, often by means of direct protein–protein interactions. The partners identified so far include polyQ binding protein-1 (PQBP1) [30], the mediator of retinoid and thyroid hormone receptors SMRT/SMRTER [28], the repressor Capicua (CIC) [31], the transcription factors Senseless/Gfi-1 [32] and Sp1 [33], transcriptional complexes like Tip60-RORα [34], LANP-E4F [35] and HDAC-MEF2 [36], and, more recently, CBF1, a Notch signaling pathway transcription factor [37]. Several of these interactions map to the AXH domain (Figure 2A) and have been shown to be positively or negatively affected by expansion of the polyQ with consequent perturbation of the associated transcriptional pathway.

In addition to a role in transcriptional regulation [10,12], Atx1 is an RNA binding protein. It recognizes RNA homo-polymers in a manner that depends on the length of the polyQ tract [38]. The AXH domain was also identified as the region responsible for RNA homo-polymer binding, exhibiting the same preference as the full-length Atx1 [16]. Further support for a role of Atx1 in RNA metabolism is provided by investigations of the protein interactome, most of which consists of proteins involved in RNA binding. Atx1 was shown to recruit the mRNA export factor TAP/NXF1 in an RNA-dependent manner, suggesting a role in processing and/or exporting specific mRNAs to the cytoplasm [39]. Interestingly, the majority of the RNA binding proteins thereafter identified as Atx1 binding partners, that is, RBM9/FOX2, A2BP/FOX-1 [40], RBM17/SPF45 and U2AF65 [41], are splicing factors. In the case of RBM17 and U2AF65, the interaction was mapped onto the short linear UHM ligand motif (ULM) in the C terminus of Atx1, a sequence known to be involved in recognition of the splicing factor U2AF (U2 auxiliary factor) homology motif (UHM) domain [20]. It was also shown that Atx1 has a positive effect on U2AF65 splicing [20] and that overexpression of Atx1 positively affects splicing of transcripts of the other polyQ-containing protein ataxin-2 by RBM9/FOX2 [42]. These findings strongly suggest a specific role of Atx1 in pre-mRNA processing.

## Aberrant aggregation: non-polyQ regions have a role in aggregation and disease

Although polyQ expansion plays a predominant role in disease, it has been demonstrated that polyQ expansion is a necessary but not sufficient condition for disease development and that regions either flanking or sequentially distant from the polyQ tract contribute to protein aggregation [12].

One such region is the AXH domain [29] and, C terminal to it, the stretch that contains phosphorylable S776 and the nuclear localization signal (NLS). These motifs seem to be particularly important for the formation of inclusions and SCA1 progression [12,15,19,20,29,43]. For example, it was demonstrated that the isolated AXH domain possesses an intrinsic ability to aggregate and that, *in vitro*, the domain spontaneously forms fibers even in the absence of destabilizing conditions, whereas in eukaryotic cells its propensity to multimerization positively influences the aggregation of the expanded full-length protein [29]. Thus, it has been suggested that, in addition to polyQ, AXH may act as a second aggregation ‘hotspot’. The possibility of forming dimers, tetramers, and higher molecular species observed at the level of the isolated AXH seems to be on-pathway to fiber formation. This could explain why overexpression of non-expanded Atx1 (i.e., 30 glutamines) in flies and mice causes phenotypes similar to those caused by overexpression of *Drosophila* Atx1, which lacks the polyQ tract, but different from those observed for polyQ peptides [27,32]. The evidence of an involvement of AXH as an independent aggregation hotspot is so compelling as to allow Zohgbi and coworkers to suggest that ‘the AXH domain but not the expanded polyQ tract could be necessary to generate the Atx1 gain-of-function phenotype in flies’ and be required for SCA1 pathogenesis [32].

Therefore, the pathological behavior of Atx1 is a consequence of a complex crosstalk between several different regions. This is not the first example of a protein with multiple aggregation sites comprising both intrinsically unfolded and globular domains [44].

## Defense mechanisms: role of protein–protein interactions in disease development

**Figure 3:**
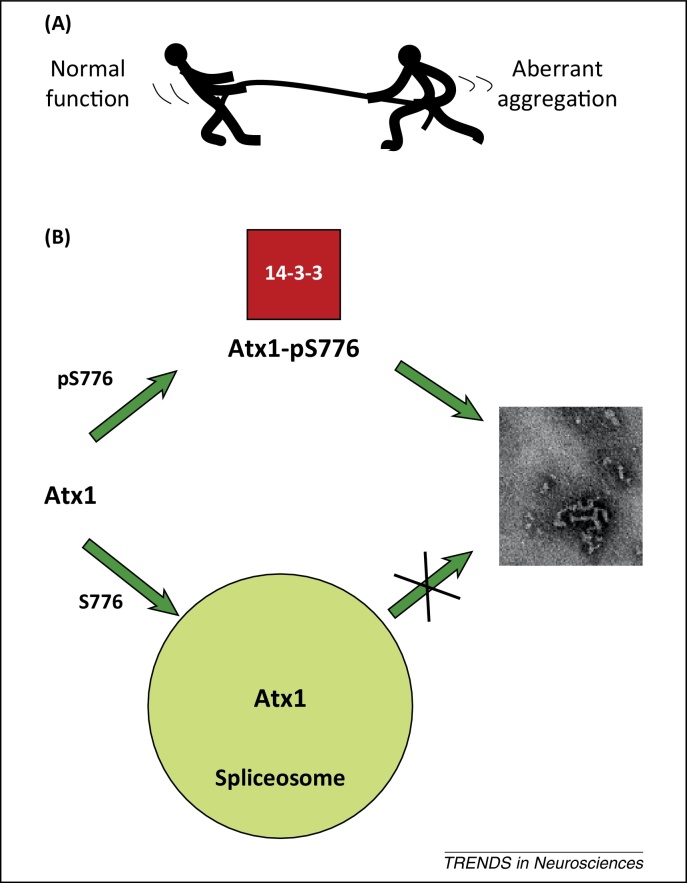
Protein–protein interaction seems to be an important element that determines protein function. **(A)** Normal function as a competing pathway to aggregation. **(B)** Schematic representation of the role of phosphorylation of S776 as a molecular switch. When S776 is non-phosphorylated, ataxin-1 (Atx1) is part of a large multiprotein complex and is likely to engage in multiple cooperative interactions with spliceosomal factors being thus protected against aggregation. When it is phosphorylated, it interacts with 14-3-3 and other such proteins and remains more prone to aggregation.

Protein aggregation has been suggested to be a competing pathway of normal function and therefore of functional protein–protein interactions [7] (Figure 3A). This statement appears to hold true for Atx1; although the subject is still debated, SCA1 appears to be better explained by a loss of function caused by alterations of native protein–protein interactions via protein aggregation than by the acquisition of novel aberrant interactions acquired through polyQ expansion [41,45]. A direct competition between interactions with other cellular partners and self-association is also in agreement with the observation that transcription dysregulation occurs already at the very early stages of the disease [25,27,46]. In this view, the characterization of native interactions of Atx1 with its cellular partners not only is relevant to identify the protein function but could also be important for understanding how interacting proteins modulate aggregation and pathogenesis.

As mentioned above, most of the known interactions of Atx1 with its cellular partners have been mapped onto the C terminus of the protein, either on the AXH domain or on short linear motifs around the phosphorylable S776. Phosphorylation of S776, in particular, acts as a molecular switch that allows the protein to discriminate between different binding partners [20] (Figure 3B). The phosphorylation state of S776, which, notably, is a residue sequence-wise very distant from polyQ, has been shown to be necessary for pathogenesis in the presence of expansion [19]. Among the interacting partners of Atx1 in this region are the molecular adaptor 14-3-3, which specifically binds S776-phosphorylated Atx1, and the splicing factors U2AF65 and SPF45, which recognize the non-phosphorylated protein [20,47]. Based on this evidence, we formulated the hypothesis that participation in interactions with SPF45 and U2AF65 that are components of the large dynamic spliceosome machine could play a protective role against self-association of expanded Atx1 [19,20,47]. This would prevent or reduce self-association of Atx1 to an extent that would allow efficient clearance by the proteasome pathway machinery. Conversely, when Atx1 is S776-phosphorylated and recruited by the relatively small 14-3-3, the interaction with the spliceosome complex is hampered and expanded Atx1 becomes available to self-association. This could be a possible mechanism of defense from aggregation.

## Can protein–protein interactions be used for therapeutic purpose?

An even more interesting example of how a cellular partner can counteract the intrinsic propensity of Atx1 towards aggregation is that of the transcriptional repressor CIC. This protein forms a stable complex with wild type Atx1 and participates in the formation of a large native complex in mouse cerebellum [31,41]. PolyQ expansion attenuates the formation of the Atx1–CIC complex and weakens the corepressive function exerted by the complex [31,41,46].

**Figure 4:**
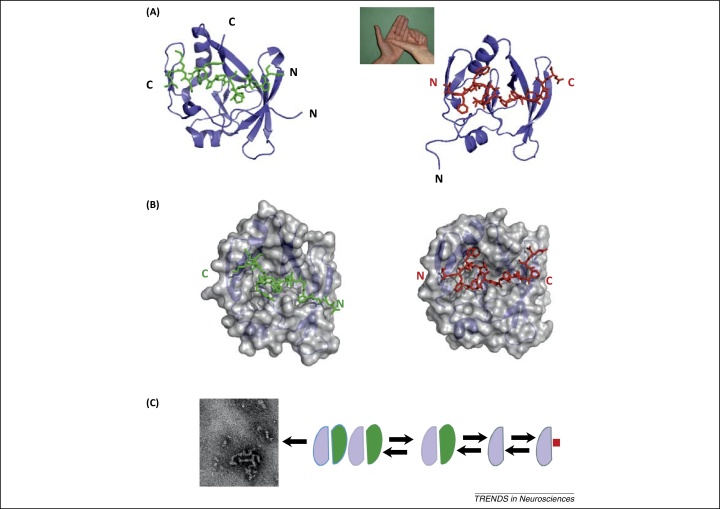
Interaction with the protein CIC suggests a possible approach to drug design. **(A)** Comparison of the structures of the AXH domain in the dimer (left panel, PDB accession code 1oa8, [21]) and of a complex with a synthetic peptide spanning the sequence of the CIC N terminus (right panel, PDB accession code 2m41[49]). The structure of the complex corresponds, in our analogy, to having the palm wide open because the N terminus is now pushed out. **(B)** The same as in (A) but representing the AXH monomers with the Van der Waals surface. The N terminus of the other protomer in the dimeric form (in green) packs in the same groove occupied by the CIC peptide (in red). The two interacting chains adopt opposite orientations. [Note that the two structures were independently solved in the crystal (the dimer) and in solution (the complex). This explains the different looking van der Waals envelope that is determined by the side chain rotamers.] **(C)** A schematic model of the equilibrium between multiple species of the AXH domain in solution and how this can relate to aggregation. The presence of CIC (shown as a red square) shifts the equilibrium towards the monomeric form thus stabilizing the protein against aggregation. Abbreviations: CIC, Capicua; PDB, Protein Data Bank.

Recognition between Atx1 and CIC has been mapped onto the AXH domain and a short linear motif located in the N terminus of CIC [31]. The structures of the AXH complexes with peptides of different lengths spanning the CIC sequence were determined both by X-ray crystallography and solution NMR spectroscopy [48,49]. In the complex, the structure of AXH changes once again, thus confirming the extraordinary structural plasticity of this chameleon protein: the N terminus of monomeric AXH no longer packs against the rest of the domain (as if the hand opened up; Figures 2C and 4A). The peptide replaces, in a reversed orientation, the N terminus of the other dimer protomer, shielding the hydrophobic core of the AXH domain from solvent exposure in a similar way as observed in the dimer (Figure 4B). This shows that the AXH domain is capable of establishing palindromic interactions.

The minimal CIC sequence necessary and sufficient to recognize AXH with nanomolar affinity was identified in the stretch V34–L44 with W37 and L40 of CIC being the only residues significantly decreasing the peptide affinity when mutated into alanine [49]. Interestingly, interaction with the CIC peptides competes with AXH dimerization, but the precise effect depends on the length of the peptide. In the crystal structure, binding of a longer CIC peptide (residues 28–48) disrupts the AXH dimer but allows another form of dimerization that is, in turn, mediated by CIC [48]. In the solution structure that was solved using the minimal CIC sequence necessary for binding with high affinity (residues 34–44) dimerization is blocked altogether (Figure 4A) [49]. This is caused by complex formation with the shorter peptide, which results in the stabilization of monomeric AXH and prevents aggregation as measured via analytical gel filtration recorded under the same conditions in which the AXH domain in the ‘free’ form aggregates spontaneously and forms amyloid-like fibers after 1 week. When the complex is formed, aggregation is hampered so completely that the complex remains monodisperse at 37°C for at least 30 days.

These results have the potential to be translated into a treatment for SCA1. If what we observe at the level of the isolated domain could be transferred to the full-length protein, the CIC sequence found to bind Atx1 with high affinity could be used directly as a mould to design compounds able to stabilize monomeric Atx1 and prevent aggregation. It would be particularly interesting to design peptidomimetic molecules based on the CIC sequence that would be more suitable to this aim than easily degradable peptides (Box 1). This strategy could result in an approach very specific to SCA1 and therefore very different from the use of generic anti-aggregation molecules, such as polyols, methylene blue, or other such compounds [2].

## Concluding remarks

Atx1 provides an interesting example of how proteins are protected from aberrant aggregation. The example stresses once again the importance of protein–protein interactions in determining and diversifying protein function. It also shows the importance of studying the nonpathological function of proteins implicated in misfolding diseases in parallel with the pathological properties. A more general understanding of their cellular role seems indeed to be essential to suggest new, specific strategies for drug development. We thus hope that the message contained in the specific example of Atx1 will be inspirational for other proteins of the misfolding disease family.

## Box 1 Outstanding questions

• **Role of Atx1 in RNA metabolism:** More experiments will be needed to assess the specific role of the protein in RNA metabolism. The current evidence supports more than one function and partner specificity in different cellular localizations [20,38–41]. New *in vivo* experiments as complemented by *in vitro* work should clarify the role of all the binding partners in a time-resolved way.
• **Gain and loss of function:** A certain degree of ambiguity, sometimes even among the same authors, remains on whether SCA1 is caused by a gain or a loss of function. It was argued that polyQ expansion favors the formation of specific protein complexes, which contribute to SCA1 through a gain-of-function mechanism. However, at the same time, it was also noted that polyQ expansion attenuates the formation of other protein complexes (for instance that with CIC), contributing to SCA1 through a partial loss-of-function mechanism [41,45]. It will thus be important to clarify this aspect further and understand the extent and circumstances that may favor one or the other mechanism.
• **Role of AXH domain in aggregation:** Although it is clear that AXH has a role in aggregation, it is still unclear whether blocking its aggregation could be beneficial for the aggregation of full-length Atx1 especially in the presence of polyQ expansion. Further studies will be required to ascertain whether the ‘protective’ effect produced by interaction with the peptide ligand from CIC is also observed in the context of the expanded full-length protein. This hypothesis could be tested, for instance, by transient co-transfection of the protein and the peptide to test whether the aggregation foci formed by Atx1 are cleared/reduced in the presence of the interaction.
• **Drug design in SCA1:** Once the role of AXH dimerization in aggregation is clarified, it will be of particular interest to design peptidomimetic molecules that mimic the features of the CIC peptides. This strategy could result in an approach specific to SCA1 that could thus be more effective than the use of generic anti-aggregation molecules, such as polyols, methylene blue, or other such compounds [2]. An even more powerful approach could be that based on structure–activity relationship (SAR) by NMR originally developed by Stephen Fesik and coworkers [50]. With this technique, compounds with higher affinities could be obtained by tethering, with a linker of suitable length, the CIC-like compound with ligands that prevent polyQ aggregation, thus obtaining even more specific drugs. At the same time, a wider screening of mutations on the peptide may be important to provide better bases for drug design.

## Glossary

Ataxia: the word comes from the Greek language and refers to a neurological loss of voluntary coordination of muscular movement. Ataxia is a nonspecific clinical manifestation that implies dysfunction of different parts of the nervous system that coordinate movement, such as the cerebellum. Ataxias can affect anyone of any age and can be both recessive or dominant.
Linear motifs: are short, conserved motifs involved in recognition and targeting functions. These motifs are linear, in that they do not involve 3D organization with sequence-wise distant segments of the molecule. Examples are phosphorylation motifs and NLSs.
Misfolding diseases: a group of diseases caused by protein aggregation and misfolding. Examples are Alzheimer's and Parkinson's diseases.
Oligonucleotide-binding (OB) fold: is a compact structural motif frequently found in proteins involved in nucleic acid recognition. Structural comparison of all OB fold/nucleic acid complexes solved to date confirms the low degree of sequence similarity among members of this family while highlighting several structural determinants common to most of these OB folds.
Polyglutamine diseases: are a family of neurodegenerative diseases caused by the anomalous expansion of a CAG triplet repeat in a specific gene. This leads to an expanded tract of glutamines in the corresponding gene products, which makes the carrier proteins highly fibrillogenic. These diseases include Huntington disease, spinobulbar muscular atrophy, dentatorubral-pallidoluysian atrophy, and several spinocerebellar ataxias.
Protein chameleon: proteins or protein regions that can adopt multiple local or global folds usually because of different environments or sequence variations. This possibility is thought to facilitate evolutionary transitions in protein structure and function. It is rather unusual to observe different conformations (asymmetric subunits) in crystal structures.
Protomer: the term used in structural biology to refer to the smallest subunit that assembles in a defined stoichiometry to form an oligomer. It is, for instance, the monomeric subunit in a dimer.
Spinocerebellar ataxias (SCAs): are progressive neurodegenerative diseases with multiple types, each of which could be considered a disease in its own right.
Structural domains: are a conserved part of a given protein sequence that can evolve, function, and exist independently of the rest of the protein chain. Each domain forms a compact 3D structure and often can be independently stable and folded.
U2AF (U2 auxiliary factor) homology motif (UHM): is an RNA recognition domain that binds to tryptophan-containing linear peptide motifs (ULMs) in several nuclear proteins.
UHM ligand motif (ULM): is a short induced fit linear motif that mediates dynamic interactions between splicing factors.
